# Selective Synthesis of 3-*O*-Palmitoyl-Silybin, a New-to-Nature Flavonolignan with Increased Protective Action against Oxidative Damages in Lipophilic Media

**DOI:** 10.3390/molecules23102594

**Published:** 2018-10-10

**Authors:** Samantha Drouet, Joël Doussot, Laurine Garros, David Mathiron, Solène Bassard, Alain Favre-Réguillon, Roland Molinié, Éric Lainé, Christophe Hano

**Affiliations:** 1 Laboratoire de Biologie des Ligneux et des Grandes Cultures, INRA USC1328, Université d’Orléans, 45067 Orléans, France; samantha.drouet@univ-orleans.fr (S.D.); joel.doussot@lecnam.net (J.D.); laurine.garros@univ-orleans.fr (L.G.); 2 Bioactifs et Cosmétiques, GDR 3711 COSMACTIFS, CNRS/Université d’Orléans, 45067 Orléans CÉDEX 2, France; 3 Département Chimie Vivant Santé (EPN 7), Conservatoire National des Arts et Métiers, 75141 Paris CEDEX 03, France; alain.favre-reguillon@lgpc.cpe.fr; 4 Institut de Chimie Organique et Analytique, ICOA UMR7311, Université d’Orléans-CNRS, 45067 Orléans CÉDEX 2, France; 5 Plateforme Analytique, Institut de Chimie de Picardie FR 3085 CNRS, Université de Picardie Jules Verne, 33 rue St Leu, 80039 Amiens, France; david.mathiron@u-picardie.fr; 6 BIOPI EA3900, Biologie des Plantes et Innovation, Université de Picardie Jules Verne, 80037 Amiens, France; solene.bassard@u-picardie.fr (S.B.); roland.molinie@u-picardie.fr (R.M.); 7 Laboratoire de Génie des Procédés Catalytiques (UMR 5285), Université de Lyon, CPE Lyon, 43 boulevard du 11 Novembre 1918, 69100 Villeurbanne, France

**Keywords:** Antioxidant, anti-lipoperoxidant, flavonolignan, palmitoylation, *Silybum marianum*

## Abstract

A selective acylation protocol using cerium chloride (CeCl_3_) as catalyst was applied to functionalize silybinin (**1**), a natural antioxidant flavonolignan from milk thistle fruit, in order to increase its solubility in lipophilic media while retaining its strong antioxidant activity. The selective esterification of **1** at the position 3-OH with a palmitate acyl chain leading to the formation of the 3-*O*-palmitoyl-silybin (**2**) was confirmed by both mass spectroscopy (MS) and nuclear magnetic resonance (NMR) analyses. The antioxidant activity of **1** was at least retained and even increased with the CUPRAC assay designed to estimate the antioxidant activity of both hydrophilic and lipophilic compounds. Finally, the 3-*O*-palmitoylation of **1**, resulting in the formation of **2**, also increased its anti-lipoperoxidant activity (i.e., inhibition of conjugated diene production) in two different lipophilic media (bulk oil and *o*/*w* emulsion) subjected to accelerated storage test.

## 1. Introduction

Lipids are essential constituents of food and cosmetic preparations but are predisposed to oxidation that constitute the major source of quality deterioration of these products and could result in important health issues for the consumers [1]. In order to avoid lipid oxidative deterioration, antioxidants are added at permissive levels in these preparations [1]. Over the last decade, natural antioxidant from plant origin have attracted growing interest in replacement of the largely used, but potentially harmful, synthetic antioxidant such as butylated hydroxyanisole (BHA) or butylated hydroxytoluene (BHT) [2,3,4].

Silybin (**1**) is the major naturally occurring flavonolignan, accumulated in large amounts in the milk thistle (*Silybum marianum* L. Gaernt) fruit, as a part of the so-called silymarin mixture [5,6]. Milk thistle has been used for centuries as a medicinal plant to cure various diseases and was used traditionally in the European pharmacopoeia as liver detoxifier as well as an antidote against *Amanita phalloides* intoxication [5,7]. Most recently, those effects have been ascribed to the pronounced anti-inflammatory actions, anti-cancer, and antioxidant activity of **1** [8,9,10,11,12]. Its effective antioxidant action have been described and molecular mechanism elucidated [6,12]. From these results, it can be assumed that (**1**) could be a serious candidate to stabilize lipophilic media against oxidative deterioration for food and cosmetic applications.

Natural **1** is composed of two stereoisomers: silybin A (2R, 3R, 10R, 11R) and silybin B (2R, 3R, 10S, 11S) and is poorly soluble in water and in lipids [12]. For this purpose, there are several examples of semisynthetic modifications to increase its bioavailability or solubility in lipophilic media while retaining its biological activity. Silybin (**1**) is already used after modifications designed to increase its water solubility as it is the case in Legalon^®^, a common drug protecting the liver where **1** is present in the form of a bis-hemisuccinate derivative. Other phosphodiester and glyco-conjugates were produced in order to increase solubility in water [13,14]. Similarly, derivatives can be made to increase the solubility in hydrophobic media, like silybin 7-*O*- and 23-*O*-acyl-derivatives of various acyl chain lengths [12]. These compounds were tested for their antioxidant through inhibition of lipid peroxidation and 2,2-diphenyl-1-picrylhydrazyl (DPPH)-scavenging assays. The palmitate esters of **1** were found to be the best radical scavengers and lipid peroxidation inhibitors [12]. However, the 3-OH acyl substitution was not achieved using this method.

The present study aimed at branching selectively at the position 3-OH of **1** an acyl C16 and evaluating the antioxidant activity of the generated 3-*O*-palmitoyl-silybin (**2**) in lipophilic media such as bulk oil and *o*/*w* emulsion.

## 2. Materials and Methods

### 2.1. Synthesis Protocol

Silybin (**1**), palmitic anhydride, CeCl_3_·7H_2_O and all chemicals were purchased from Sigma Aldrich (Saint-Quentin Fallavier, France). The reaction was monitored by thin-layer chromatography (TLC) on F_254_ silica gel (Merck Millipore Fontenay sous Bois, Paris, France) and the spots were visualized with a UV light. 

In a 50 mL flask equipped with magnetic stirring, palmitic anhydride (0.40 mmol), and CeCl_3_·7H_2_O (0.10 mmol) were added with 20 mL of THF. The mixture was kept under vigorous stirring for 30 min. Then, a solution of **1** (0.40 mmol) in 20 mL of THF was added dropwise and the reaction was stirred for 24 h at room temperature. After completion, the solvent was evaporated under reduced pressure and the crude product was solubilized in the minimum volume of CH_2_Cl_2_ and purified by column chromatography on silica gel (Grade 633, 200–425 mesh). Elution with CH_2_Cl_2_/ethanol (97/3) afforded **2** (Rf: 0.34) as a white amorphous solid with 66% of yield and 25% of unreacted **1** (Rf: 0.25) after elution with CH_2_Cl_2_/ethanol (80/20).

### 2.2. Electrospray High Resolution Mass Spectrometry (ESI-HRMS) Study

A methanolic solution of the purified compound **2** (0.1 mg/mL) was analyzed by ESI-HRMS and by tandem mass spectrometry (MS/MS) in the negative ionization mode. Flow injection analysis was performed using an ACQUITY UPLC H-Class system (Waters, Manchester, UK) coupled with a Synapt G2-Si Q-TOF hybrid quadrupole time-of-flight instrument (Waters, Manchester, UK) equipped with an electrospray (ESI) ionization source (Z-spray) and an additional sprayer for the reference compound (Lock Spray). The injection volume was 1 μL and a methanol flow rate of 0.4 mL/min fully directed toward the ESI source of the QTof instrument was used. The source and desolvation temperatures were 100 °C and 250 °C, respectively. Nitrogen was used as a drying and nebulizing gas at flow rates of 50 and 500 L/h, respectively. The capillary voltage was 2 kV, the sampling cone voltage 20 V and the source offset 20 V. Lock mass corrections using [M-H]^−^ at *m*/*z* 554.2615 of a leucine-enkephalin solution (1 ng/μL in 50:50 acetonitrile/water + 0.1% formic acid) were applied for accurate mass measurements and elemental composition determination. The mass range in full-scan mode was 50–2000 Da and spectra were recorded at 0.2 s/scan in the profile mode at a resolution of 25,000 (FWHM). For MS/MS experiments, argon was used as collision gas and the collision energy was optimized to 33 eV for [M-H]^−^ precursor ion at *m*/*z* 719. Data acquisition and processing were performed with MassLynx 4.1 software.

### 2.3. NMR Analysis

For nuclear magnetic resonance (NMR) analysis, compounds were dissolved in 0.75 mL of DMSO-*d*_6_ containing 0.03% of tetramethylsilane (TMS). NMR analysis protocols were adapted from previous works [15]. Briefly, ^1^H-NMR spectra were obtained using a classic proton sequence (90° proton pulse), 12,019 Hz spectral widths and 2 s relaxation delay. Each spectrum consisted of 4 dummy scans and 512 scans of 128 K data points. The NMR signal was multiplied by an exponential weighing function corresponding to a line broadening of 0.3 Hz prior to Fourier transformation. The DEPTQ spectra were acquired using 8 dummy scans and 24 K scans of 64 K data points, using spectral widths of 37,878 Hz. A 2 s relaxation delay was employed. To obtain a better ^13^C sensitivity in particular to enhance the intensity of quaternary carbons, UDEFT experiment has been performed using a 5 mm BBFO probe. The UDEFT spectra were acquired using 4 dummy scans and 24 K scans of 32 K data points, using spectral widths of 37,878 Hz. A 2s relaxation delay was employed. The FID was multiplied by an exponential weighing function corresponding to a line broadening of 2 Hz prior to Fourier transformation. The 2D COSY spectra were acquired using 8 scans per 256 increments that were collected into 2 K data points, using spectral widths of 12,019 Hz in both dimensions. A 2 s relaxation delay was employed. The 2D TOCSY spectra were acquired using 8 scans per 256 increments that were collected into 2 K data points, using spectral widths of 12,019 Hz in both dimensions. A 2.0 s relaxation delay and a mixing time of 100 ms were employed. The 2D HSQC spectra were acquired with 2 s relaxation delay using 8 scans per 256 increments that were collected into 4K data points, using spectral widths of 12,019 Hz in F2 and 26,412 Hz in F1. The 2D HMBC spectra were acquired with 2 s relaxation delay using 16 scans per 256 increments that were collected into 4K data points, using spectral widths of 12,019 Hz in F2 and 37,732 Hz in F1. All non-zero filled obtained spectra were manually phased and baseline-corrected, calibrated at 0.0 ppm to TMS.

### 2.4. Determination of Antioxidant Activity

Ferric reducing antioxidant power (FRAP) [16] and cupric ion reducing antioxidant capacity (CUPRAC) [17] protocols were used to evaluate the in vitro antioxidant activity of the compounds. Briefly, 10 μL of the extracted sample was mixed with 190 μL of FRAP (10 mM TPTZ; 20 mM FeCl_3_·6H_2_O and 300 mM acetate buffer pH 3.6; ratio 1:1:10 (*v*/*v*/*v*)) were used for FRAP assay, whereas 10 μL of the extracted sample was mixed with 190 μL of CUPRAC (10 mM Cu(II); 7.5 mM neocuproine and 1 M acetate buffer pH 7; ratio 1:1:1 (*v*/*v*/*v*)) were used for CUPRAC assay. Incubation lasted 15 min at room temperature. Absorbance of the reaction mixture was measured at 630 nm for FRAP and 450 nm for CUPRAC with a BioTek ELX800 Absorbance Microplate Reader (BioTek Instruments, Colmar, France). Assays were made in triplicate and antioxidant capacity was expressed as Trolox C equivalent antioxidant capacity (TAEC).

### 2.5. Linseed Bulk Oil and Preparation of o/w Emulsion

Organic refined cold pressed linseed oil free of synthetic antioxidant was obtained from D.R.P. organic farming (Vicq Exemplet, France) and was used as bulk oil. A 70% *v*/*v* linseed oil-derived *o*/*w* emulsion was prepared as described previously by Hano et al. (2017) [3].

### 2.6. Accelerated Degradation Protocol

Triplicate samples of refined cold pressed milk thistle seed oil and *o*/*w* emulsion were prepared as follows: 555 mmoles per kg, corresponding to half the permitted synthetic antioxidant concentration in oil defined by the Codex Alimentarius, was used for **1** and **2** as well as for the commercial antioxidants ascorbic acid and ascorbyl palmitate (Sigma Aldrich, Saint-Quentin Fallavier, France). All compounds were dissolved in DMSO at 1% of the final oily solution. DMSO at 1% of the final was added to the control sample. Accelerated degradation was performed according to the Schaal oven test: Samples (20 g) were placed in open tinted amber glass vials (100 mL, 4 cm in diameter) in a ventilated oven set at 65 °C (one day is considered to be equivalent to 1 month of storage at room temperature). Bulk oil and *o*/*w* emulsion samples were sampled at day 6 to determine the influence of antioxidant addition on the oxidative stability of the corresponding oily preparations and stored at −30 °C until analysis.

### 2.7. Determination of Inhibition of Conjugated Diene Hydroperoxides (CD) Formation

Conjugated diene hydroperoxides (CD) were determined using the method described by Iqbal (2007) [18]: 10mg of oily samples were dissolved in 5 mL cyclohexane and the absorbance of the resulting extract was measured at 234 nm (JASCO spectrophotometer, Lisses, France). Inhibition of CD production was expressed in percentage relative to the control conditions.

### 2.8. Statistical Analysis

All data presented are the means and standard deviations of at least three independent replicates. All statistical analyses were performed using XL-STAT 2017 software (Addinsoft, Paris, France). The sample means were distinguished using the Student Newman-Keuls method (*p* < 0.05).

## 3. Results and Discussion

### 3.1. Selective Synthesis of 3-O-palmitoyl-silybin

**Figure 1:**
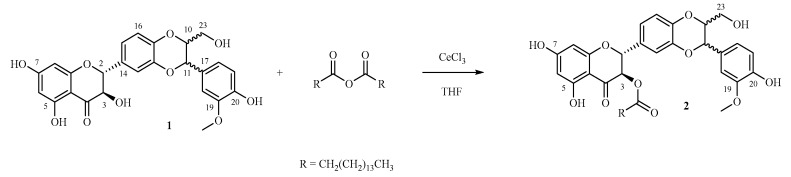
Scheme of the selective synthesis of **2**.

The selective acylation of silybin (**1**) (PubChem CID 31553) in position 3-OH to give 3-*O*-palmitoyl-silybin (**2**) was accomplished with the use of palmitic anhydride in the presence of the very oxophilic Lewis acid CeCl_3_·7H_2_O (Figure 1). This approach was inspired by the work of Gilles et al. (2015) [19] that prepared several alkyl esters of juglone in presence of this Lewis acid and with fatty acids and *N*,*N*′-dicyclohexylcarbodiimide/*N*,*N*-dimethylpyridin-4-amine(DCC/DMAP) as in Steglich esterification.

The yield, around 66%, was satisfactory, as a comparison when performing palmitate addition in position 7-OH, Gažák et al. [12] reported yields around 30%. The purity was evaluated by using H-2 signal of **2** versus other residual H-2 signals (Supplementary Figure S2). About 85% of palmitate was found linked in position 3-OH and only 15% in position 5-OH making it not able to decrease the ability to exert its antioxidant activity. The selectivity observed certain results from the very mild operating conditions (ambient temperature, neutral medium) and the exaltation of the electrophilicity of the anhydride by complexation as proposed by Gilles et al. (2015) [19]. Gažák et al. [12], using other catalysts, produced mainly **1** functionalized with palmitate in position 7-OH or 23-OH. Therefore, this is the first report on the selective acylation of **1** with palmitate in position 3-OH.

### 3.2. Structural Confirmation

In a first approach, to confirm that the acylation reaction of compound **1** to give **2** succeeded, Electrospray ionization with high-resolution mass spectrometry (ESI-HRMS) in negative mode was used. On a full-scan MS spectrum, a [M-H]^−^ at *m*/*z* 719 has been highlighted and whose accurate mass measurement at *m*/*z* 719.3444 led to the formula C_41_H_51_O_11_ corresponding to the linkage of the C_16_ acyl chain on **1** structure.

**Figure 2:**
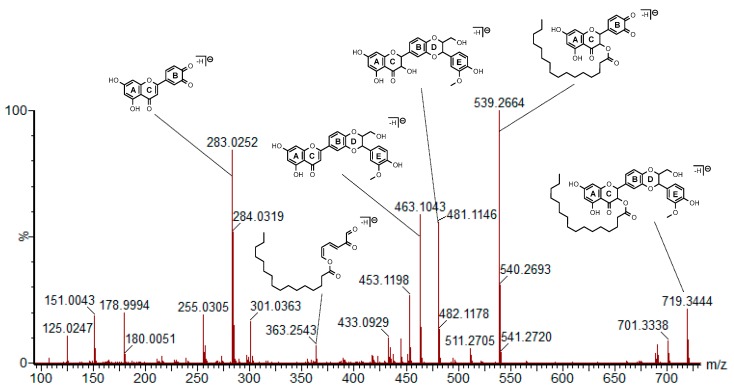
Electrospray ionization with high-resolution mass spectrometry (ESI-HRMS/MS) spectrum of the [M-H]^−^ precursor ion at *m*/*z* 719 corresponding to **2** recorded at 33 eV with hypothetic structures for the main fragments.

To gain deeper structural characterization, MS/MS experiment was performed on [M-H]^−^ at *m*/*z* 719.3444 to obtain fragments that could give information on the position of C_16_ acyl chain linkage onto **1** (Figure 2). Based on the elemental composition of fragment ions, a schematic fragmentation pathway has been proposed for the most important fragments in terms of intensity and of structural information (Supplementary Figure S1). 

From the precursor ion at *m*/*z* 719, the fragment ion at *m*/*z* 539 can be explained by a Retro-Diels-Alder (RDA) reaction in D-ring (Figure 2). 

It should be pointed out that this fragmentation mechanism is typically observed in flavonoids and their derivatives [20]. The presence of the ion at *m*/*z* 539 clearly evidences that C_16_ acyl chain is not bonded to hydroxyls from D and E rings, but is rather grafted on flavonol A-C rings. On the other hand, ions at *m*/*z* 481 and 463 can be explained by rearrangements with four-membered transition state from the parent ion at *m*/*z* 719 involving the migration of a hydrogen and respectively an elimination of C_16_H_30_O (−238 Da) and C_16_H_32_O_2_ (−256 Da). Therefore, these losses can be considered as indirect evidence of the C_16_ acyl chain linkage on **1**. Finally, a low intensity fragment ion at *m*/*z* 363 seems to be of great interest because its formula C_22_H_35_O_4_ is really apart from the other fragments by containing a high hydrogen/carbon ratio that probably comes from C_16_ acyl chain. As a hypothesis, we could propose its formation from the ion at *m*/*z* 539 by a RDA rearrangement in C-cycle and a cleavage in B-ring indicating rather that the substitution of **1** by the C_16_ acyl chain could occur on OH in position 3.

To support our MS/MS results and conclude unambiguously on the regioselectivity of the acylation reaction, a complete NMR study has been performed. First, 1D and 2D NMR experiments were carried out to obtain reference spectra of silybin (**1**), which were found to be in agreement with the literature data [12]. To highlight all OH signals for compounds **1** and **2**, ^1^H NMR spectra have been recorded in DMSO-*d*_6_ (Table 1, Supplementary Figure S2). By comparing ^1^H NMR spectra of **1** and **2**, we observed the loss of 3-OH signal on **2** providing the first hint of the acylation site (Supplementary Figure S3). Moreover, downfield chemical shift variations were observed for H-2 (5.08 to 5.50) and for H-3 (4.60 to 5.92) which can be explained by the grafting of the acyl chain on 3 OH position. As the H-3 signal became overlapped with H-6 and H-8 in **2** after the grafting of the acyl chain, the resolving power of HSQC confirmed the assignment of H3 and also allowed to observe its chemical shift variation compared to **1** (Supplementary Figure S4). The HMBC long range correlation (Supplementary Figure S5) between the proton δ 5.95 ppm (d, *J* = 11.8 Hz, H-3) and the carbon δ 171.4 ppm (C-1′) clearly evidences the formation of palmitate ester of silybin on position 3 (Supplementary Figure S6). Besides, the compound **2** exhibits a coupling constant ^3^*J*_H2-H3_ at 11.8 Hz remaining unchanged compared to **1** revealing the preservation of the trans stereochemistry after the acylation reaction. Thus, all these NMR experiments enabled to unambiguously confirm the structure of the palmitate ester of silybin in position 3. 

### 3.3. Radical Scavenging and Anti-Lipoperoxidation Activities

The antioxidant capacity of **1** and **2** were assayed using the in vitro ferric reducing antioxidant power (FRAP) and copper reducing antioxidant capacity (CUPRAC) assays and compared with commercially used antioxidants: the natural antioxidant ascorbic acid (PubChem CID 54670067) and its lipophilic counterpart ascorbyl-palmitate (PubChem CID54676825, already used as antioxidant food additive under the E number E304), and the synthetic antioxidant BHA (PubChem CID: 24667) (Table 2). The FRAP method is used to study the antioxidant activity of the hydrophilic antioxidants while the CUPRAC evaluates both lipophilic and hydrophilic antioxidants [21]. These two assays therefore make possible to observe slight variations among these different lipophilic vs hydrophilic behaviors. Here, the results clearly showed that the acylation of **1** in position 3-OH, leading to **2**, have modified its lipophilicity while retaining its high radical scavenging activity. Indeed, the radical scavenging potential of **1** was 1.74 times more effective than **2** with FRAP assay, whereas **2** was 1.55 times more effective than **1** with the CUPRAC assay (Table 2). 

The calculated Log K_ow_, respectively of 2.58 for **1** and 10.72 for **2** (Supplementary Table S1), could partly explain the different behavior observed with these two antioxidant assays. Note that the same behavior was observed with ascorbic acid and its lipophilic counterpart ascorbyl-palmitate (with Log K_ow_ of −2.41 and 6.06 respectively). Compare with classical antioxidants, the radical scavenging activity of **2** was in the range of the activities observed for ascorbic acid, ascorbyl-palmitate and BHA with the FRAP assay, whereas it was clearly better with the CUPRAC assay (Table 2), evidencing the great potential of this new-to-nature flavonolignan.

To confirm this trend, we next evaluated the anti-lipoperoxidation activities (inhibition of conjugated diene hydroperoxides (CD) formation) of **2** in two different lipophilic media (i.e., bulk oil and *o*/*w* emulsion) subjected during six days to the Schaal oven test (Table 3). All the tested antioxidants were added at 555 µmole per kg oil, corresponding to half of the permitted addition of antioxidant in refined oil defined by the Codex Alimentarius. For this purpose, an organic refined cold pressed linseed oil free of synthetic antioxidant was used in the form of bulk oil and *o*/*w* emulsion. Indeed, linseed oil rich in polyunsaturated fatty acids (PUFA) [22] and therefore particularly sensitive to oxidation is an excellent model to study inhibition of CD formation under the Schaal oven test. 

In our hands, all tested antioxidants showed an effective protection against CD formation in both lipophilic media with a prominent anti-lipoperoxidation observed for **2** (Table 3). The ability to inhibit CD formation in bulk oil was: **2** > **1** > ascorbyl palmitate > ascorbic acid = BHA, whereas in *o*/*w* emulsion it was: **2** > ascorbyl palmitate = **1** > BHA > ascorbic acid, shedding light on the interest of **2** as a new-to-nature flavonolignan with enhanced lipophilicity and increased antilipoperoxidation action for food and cosmetic applications.

Our results also showed that the lipophilic **2** was more efficient to inhibit CD formation in *o*/*w* emulsion whereas its more polar counterpart **1** was more efficient in bulk oil. This observation was in agreement with the polar paradox theory rationalizing the fact that more hydrophilic antioxidants generally appeared more effective in lipophilic media such as bulk oil, while lipophilic antioxidants displayed a higher protective action against lipoperoxidation in more polar media such as *o*/*w* emulsion [23]. This paradigm was supported by experimental data showing for examples that the lipophilic antioxidants α-tocopherol or ascorbyl-palmitate were more effective in an *o*/*w* emulsion while the more hydrophilic Trolox C and ascorbic acid have a more pronounced antioxidant action in bulk oil [24]. Here our results obtained for ascorbic acid and ascorbyl-palmitate were in agreement with this paradigm and experimental [23,24]. Under the conditions used in the present study, a significant linear relation linking Log Kow and the radical scavenging activity determined with the CUPRAC assay and the CD formation inhibition in both bulk oil and *o*/*w* emulsion was observed (Supplementary Figure S7). More recently, this paradigm was revisited and it was proposed that more complex parameters than just polarity such as the molecular size, the critical concentration or the concentration and nature of the emulsifiers used could also greatly influence the antioxidant behavior [25]. Future researches will focus on the influences of these different parameters on the antioxidant action of **2**. 

## 4. Conclusions

In this study, we have developed an efficient approach for the selective synthesis of 3-*O*-palmitoyl-silybin (**2**). The advantage of this de novo synthetized compound over the silybin (**1**) is its stronger antioxidant protective effect in hydrophobic media. We anticipate that the availability of such novel natural product analogues able to protect lipophilic media, bulk oil or *o*/*w* emulsion, against oxidative damages would be of great interest for practical applications in cosmetic and food industries.

## Figures and Tables

**Table 1:** ^1^H NMR spectral data of **2**.

NO	Proton	C
**2**	5.50 d (11.8)	79.8
**3**	5.95 d (11.8)	71.6
**4**	-	191.4
**4a**	-	101.1
**5**	-	163.2
**6**	5.95 d (2.1)	96.4
**7**	-	167.4
**8**	5.92 d (2.1)	95.4
**8a**	-	162.5
**10**	4.15 ddd (7.5, 4.8, 2.7)	78.2
	4.12 ddd (7.5, 4.8, 2.7)	78.1
**11**	4.89 d (7.9)	75.7
	4.88 d (7.9)	75.7
**12a**	-	144.1
**13**	7.12 dd (1.7, 2.2)	116.6
**14**	-	128.5
**15**	7.01 dd (8.2, 2.2)	121
	7.00 dd (8.2, 2.2)	121
**16**	6.96 dd (8.2, 1.5)	116.5
**16a**	-	143.9
**17**	-	127.4
**18**	6.98 d (1.8)	111.6
	6.99 d (1.8)	111.6
**19**	-	147.7
**20**	-	150.9
**21**	6.80 d (8.2)	115.2
	6.79 d (8.2)	115.2
**22**	6.85 d (8.2, 1.8)	120.3
	6.83 d (8.2, 1.8)	120.3
**23d**	3.54 nd	60
**23u**	nd (water signal)	60
**19-Me**	3.78 s	55.7
	3.77 s	55.7
**5-OH**	11.94 s	-
**7-OH**	11.03 br s	-
**20-OH**	9.15 s	-
**23-OH**	4.95 t (5.1)	-

**Table 2:** Radical scavenging activity evaluation

	CUPRAC *	FRAP *
Silybin	2.25 ± 0.09 ^a^	3.25 ± 0.76 ^c^
Silybinyl palmitate	3.50 ± 0.37 ^c^	1.86 ± 0.28 ^a,b^
Ascorbic acid	2.18 ± 0.19 ^b^	2.53 ± 0.18 ^b^
Ascorbyl palmitate	3.09 ± 0.09 ^b,c^	1.75 ± 0.18 ^a^
BHA	2.55 ± 0.14 ^a^	1.78 ± 0.32 ^a,b^

* expressed in Trolox C equivalent antioxidant capacity (TEAC in mM); values are the mean ± SD of 3 independent replicates; the same superscript letter indicates that the mean values are not significantly different (*p* > 0.05).

**Table 3:** Inhibition of conjugated dienes (CD) production in linseed bulk oil and *o*/*w* emulsion submitted to accelerated storage during 12 days.

Compound ^1^	Linseed Bulk Oil ^2^	*o*/*w* Emulsion ^2^
Silybin	56.83 ± 0.45 ^c^	51.90 ± 0.80 ^c^
Silybinyl palmitate	59.32 ± 0.42 ^d^	64.04 ± 0.31 ^d^
Ascorbic acid	32.51 ± 2.57 ^a^	26.73 ± 2.48 ^a^
Ascorbyl palmitate	41.67 ± 1.23 ^b^	53.11 ± 2.89 ^c^
BHA	29.06 ± 3.27 ^a^	43.34 ± 1.75 ^b^

^1^ at 500 µM final concentration; ^2^ inhibition percentage of CD production relative to control linseed bulk oil and *o*/*w* emulsion; values are the mean ± SD of 3 independent replicates; the same superscript letter indicates that the mean values are not significantly different (*p* > 0.05).

## Supplementary Materials

Supplementary Materials are available online.
