# Supplementary material for: Selective Synthesis of 3-O-Palmitoyl-Silybin, a New-to-Nature Flavonolignan with Increased Protective Action against Oxidative Damages in Lipophilic Media
Source: Molecules. 2018 Oct 10;23(10):2594. doi: 10.3390/molecules23102594 (PMC6222644; doi:10.3390/molecules23102594)
Supplement: Supplementary file 1 [file molecules-23-02594-s001.pdf]

molecules

Supplementary Materials

Synthesis of 3-O-palmitoyl-silybin, a new-to-nature antioxidant flavonolignan with increased protective action against oxidative damages in lipophilic media

Samantha Drouet[12§], Joël Doussot[123§], Laurine Garros[124], David Mathiron[5], Solène Bassard[6], Alain Favre-Réguillon[7], Roland Molinié[6], Éric Lainé[12]*, Christophe Hano[12]*

[1] Laboratoire de Biologie des Ligneux et des Grandes Cultures, INRA USC1328, Université d'Orléans, 45067 Orléans, France; samantha.drouet@univ-orleans.fr; laurine.garros@univ-orleans.fr; eric.laine@univ-orleans.fr; christophe.hano@univ-orleans.fr

[2] Bioactifs et Cosmétiques, GDR 3711 COSMACTIFS, CNRS/Université d'Orléans, 45067 Orléans CÉDEX 2, France

[3] Ecole Sciences industrielles et technologiques de l'information (SITI), Département Chimie Alimentation Santé Environnement Risque (CASER), Le CNAM 75141 Paris Cedex 03, France; joel.doussot@lecnam.net

[4] Institut de Chimie Organique et Analytique, ICOA UMR7311, Université d'Orléans-CNRS, 45067 Orléans CÉDEX 2, France

[5] Plateforme Analytique, Institut de Chimie de Picardie FR 3085 CNRS, Université de Picardie Jules Verne, 33 rue St Leu 80039 Amiens, France; david.mathiron@u-picardie.fr

[6] BIOPI EA3900, Biologie des Plantes et Innovation, Université de Picardie Jules Verne, 80037 Amiens, France; solene.bassard@u-picardie.fr; roland.molinie@u-picardie.fr

[7] Université de Lyon, Laboratoire de Génie des Procédés Catalytiques (UMR 5285), CPE Lyon, 43 boulevard du 11 Novembre 1918, 69100 Villeurbanne, France; alain.favre-reguillon@lgpc.cpe.fr

[§] These two authors contributed equally to this work and should be considered both as first authors

* Correspondence: christophe.hano@univ-orleans.fr; Tel.: +33 237 309 753

Table S1. *in silico* evaluation of Log K_{ow}

Figure S1. Proposed fragmentation pathway based on MS/MS of $[M-H]^-$ ion at m/z 719 corresponding to compound 2

Figure S2. ^{1}H NMR spectrum of 3-O-palmitoylsilybin 2 (600 MHz, DMSO-d_6, 300K)

Figure S3. Partial ^{1}H NMR spectrum of silybin (a) and 3-O-palmitoylsilybin (b) (600 MHz, DMSO-d_6, 300K)

Figure S4. Partial HSQC spectrum of 3-O-palmitoylsilybin (**2**) (600 MHz, DMSO-d_6, 300K)

Figure S5. Partial HMBC spectrum of 3-O-palmitoylsilybin (**2**) with H3/C1' long range correlation highlighted by the red box (600 MHz, DMSO-d_6, 300K)

Figure S6. ^{13}C NMR (DEPTQ) spectrum of 3-O-palmitoylsilybin **2** (150 MHz, DMSO-d_6, 300K)

Figure S7. Pearson correlation analysis linking Log K_{ow} to CUPRAC assay, FRAP assay and CD production inhibition in bulk oil and o/w emulsion

Table S1. *in silico* evaluation of Log K_{ow}

	Log K_{ow} *
Silybin	2.58 ± 0.59
Silybinyl palmitate	10.72 ± 0.60
Ascorbic acid	-2.41 ± 0.45
Ascorbyl palmitate	6.06 ± 0.59

* evaluated *in silico* using ACD/LogP DB; Note that according to PubChem, Log K_{ow} for BHA is equal to 3.06

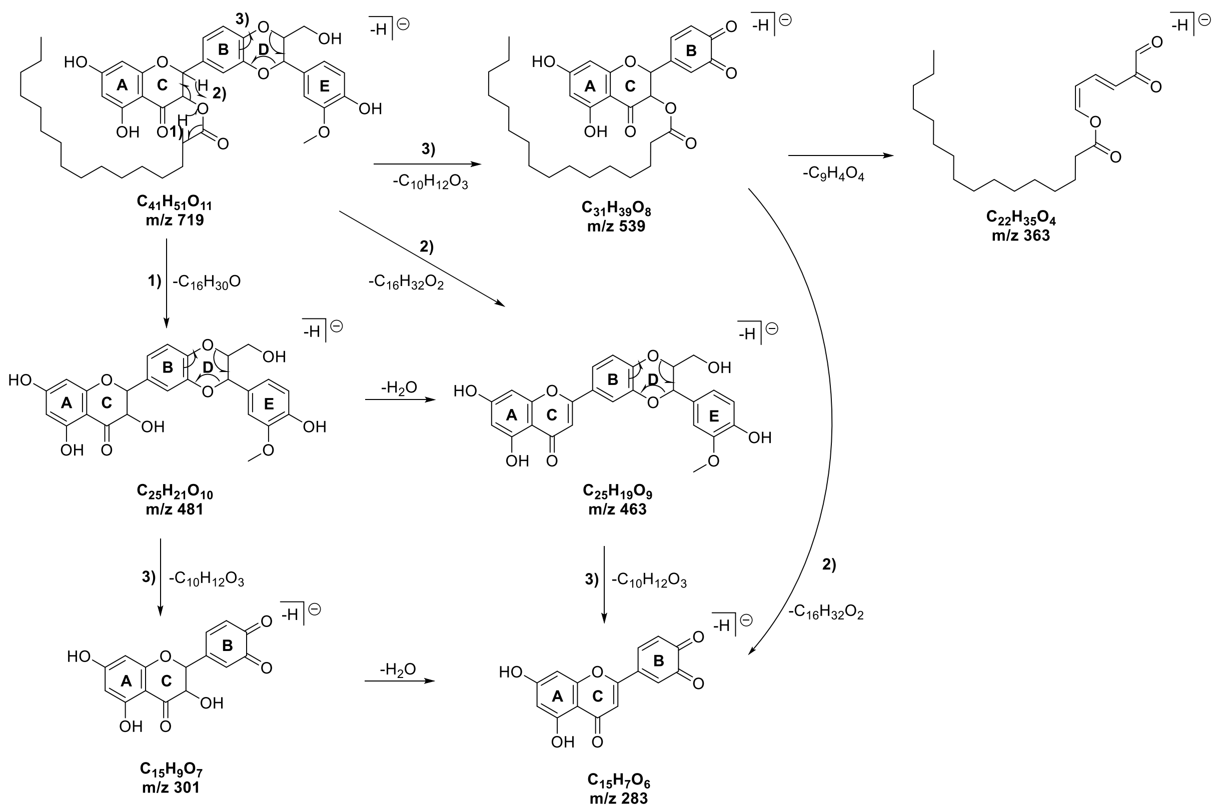

Figure S1. Proposed fragmentation pathway based on MS/MS of [M-H]$^-$ ion at m/z 719 corresponding to compound **2**

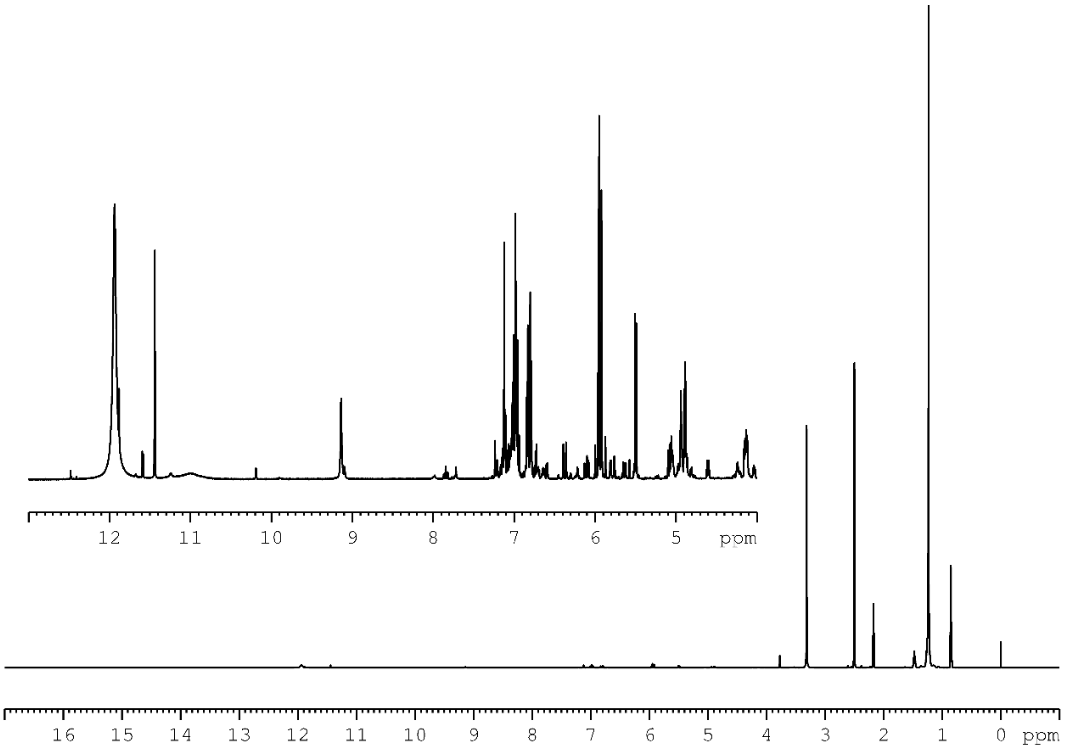

Figure S2. ^{1}H NMR spectrum of 3-O-palmitoylsilybin 2 (600 MHz, DMSO-d_6, 300K)

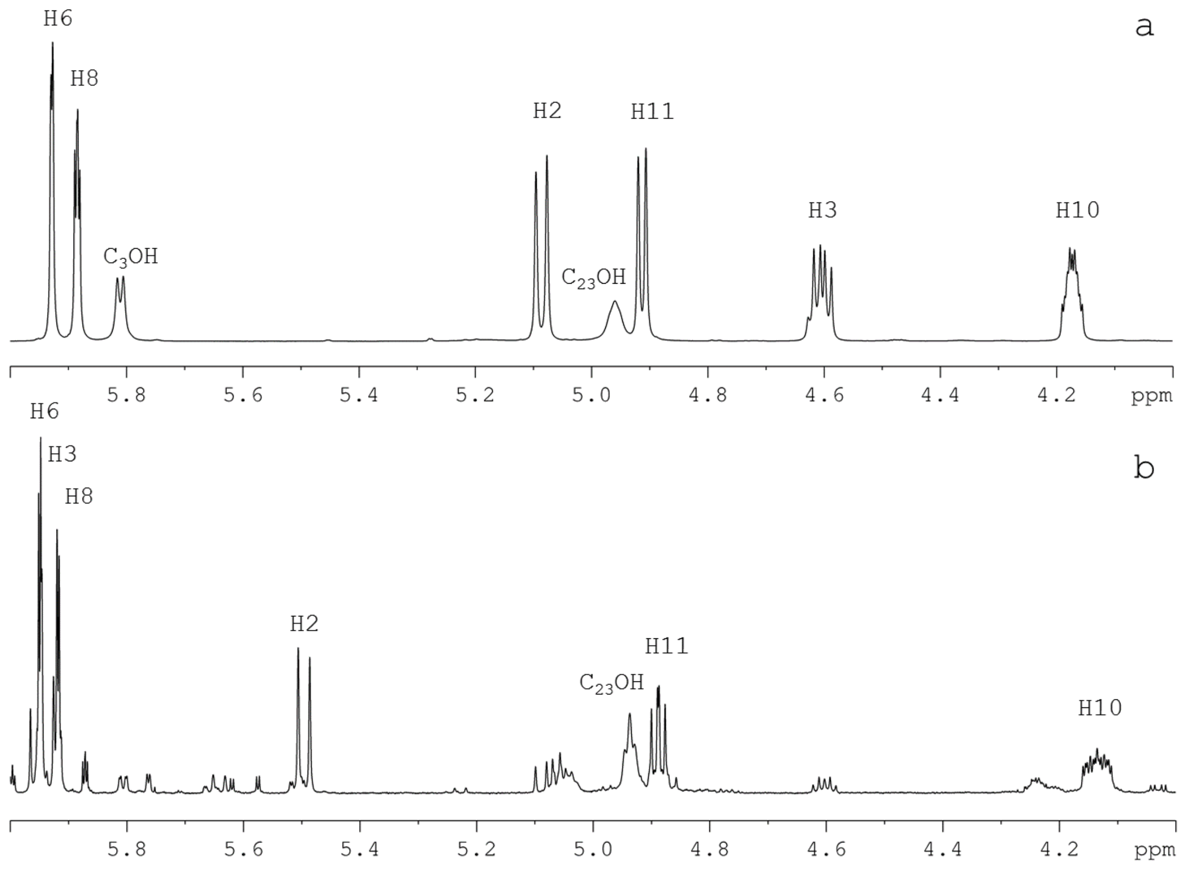

Figure S3. Partial ^{1}H NMR spectrum of silybin (a) and 3-O-palmitoylsilybin (b) (600 MHz, DMSO-d_6, 300K)

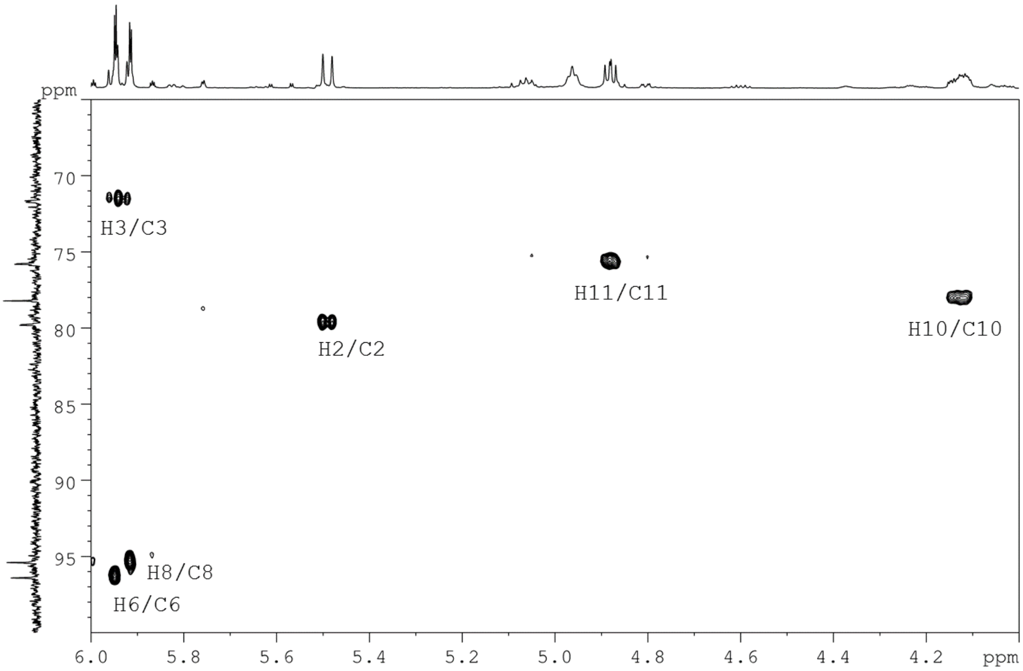

Figure S4. Partial HSQC spectrum of 3-O-palmitoylsilybin (**2**) (600 MHz, DMSO-d_6, 300K)

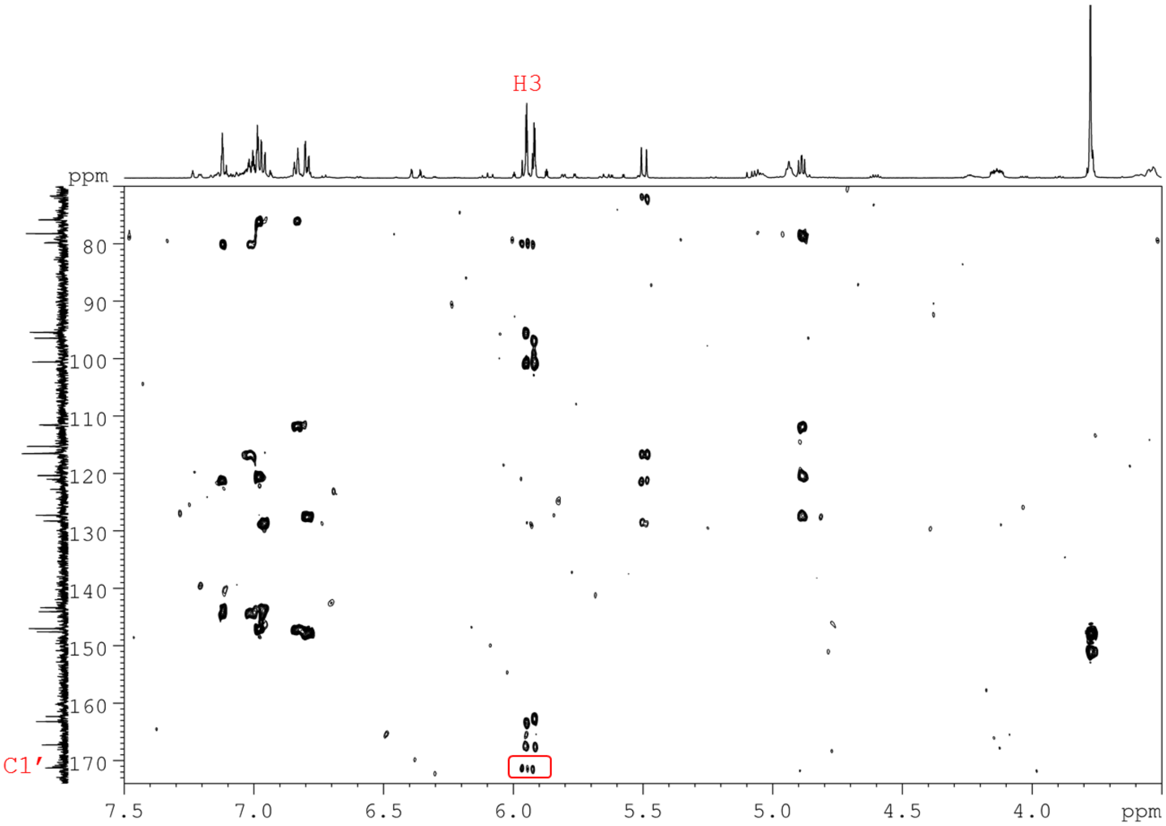

Figure S5. Partial HMBC spectrum of 3-O-palmitoylsilybin (**2**) with H3/C1’ long range correlation highlighted by the red box (600 MHz, DMSO-d_6, 300K)

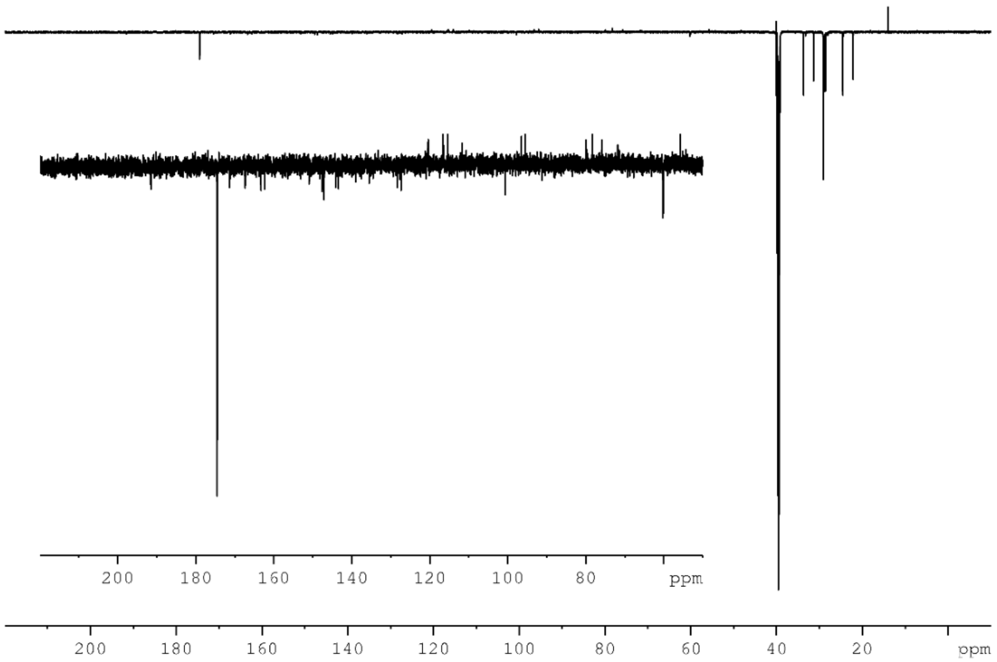

Figure S6. ^{13}C NMR (DEPTQ) spectrum of 3-O-palmitoylsilybin **2** (150 MHz, DMSO-d6, 300K)

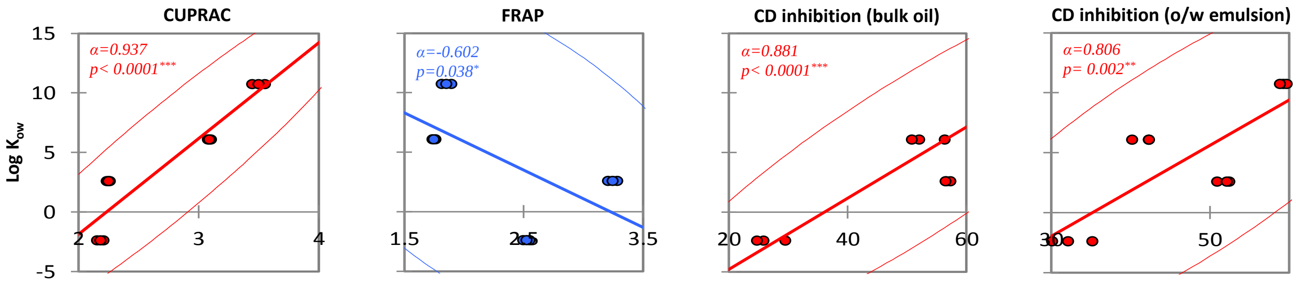

Figure S7. Pearson correlation analysis linking Log K_{ow} to CUPRAC and FRAP assays, and CD production inhibition in bulk oil and o/w emulsion

© 2018 by the authors. Submitted for possible open access publication under the terms and conditions of the Creative Commons Attribution (CC BY) license (http://creativecommons.org/licenses/by/4.0/).
